# Genomic and Transcriptomic Diversification of Flagellin Genes Provides Insight into Environmental Adaptation and Phylogeographic Characteristics in *Aeromonas hydrophila*

**DOI:** 10.1007/s00248-024-02373-4

**Published:** 2024-05-02

**Authors:** HyeongJin Roh, Dhamotharan Kannimuthu

**Affiliations:** Pathogen Transmission and Disease Research Group, Institute of Marine Research, PO Box 1870 Nordnes 5870, Bergen, Norway

**Keywords:** Bacterial flagellin, Microbial adaptation, *A. hydrophila*, Multi-omics analysis, Motility

## Abstract

*Aeromonas hydrophila* is an opportunistic motile pathogen with a broad host range, infecting both terrestrial and aquatic animals. Environmental and geographical conditions exert selective pressure on both geno- and phenotypes of pathogens. Flagellin, directly exposed to external environments and containing important immunogenic epitopes, may display significant variability in response to external conditions. In this study, we conducted a comparative analysis of ~  150 *A. hydrophila* genomes, leading to the identification of six subunits of the flagellin gene (*fla-1* to *fla-4*, *flaA*, and *flaB*). Individual strains harbored different composition of flagellin subunits and copies. The composition of subunits showed distinct patterns depending on environmental sources. Strains from aquatic environments were mainly comprised of *fla-1* to *fla-4* subunits, while terrestrial strains predominated in groups harboring *flaA* and *flaB* subunits. Each flagellin showed varying levels of expression, with *flaA* and *flaB* demonstrating significantly higher expression compared to others. One of the chemotaxis pathways that control flagellin movement through a two-component system was significantly upregulated in *flaA*(+ 1)/*flaB*(+ 1) group, whereas *flaA* and *flaB* showed different transcriptomic expressions. The genes positively correlated with *flaA* expression were relevant to biofilm formation and bacterial chemotaxis, but *flaB* showed a negative correlation with the genes in ABC transporters and quorum sensing pathway. However, the expression patterns of *fla-2* to *fla-4* were identical. This suggests various types of flagellin subunits may have different biological functions. The composition and expression levels of flagellin subunits could provide valuable insights into the adaptation of *A. hydrophila* and the differences among strains in response to various external environments.

**Supplementary Information:**

The online version contains supplementary material available at 10.1007/s00248-024-02373-4.

## Introduction

*Aeromonas hydrophila* is a gram-negative facultative anaerobic bacterium. It has been isolated from diverse environmental habitats (e.g., drinking water, wastewater, sewage, food, milk, soil) and has a broad host range from fish to humans [1–4]. *A.* *hydrophila* causes infectious disease with severe clinical signs such as gastroenteritis, necrotizing fasciitis, exophthalmia, fin rot, dropsy, hemorrhage, and septicemia as opportunistic bacterial pathogens in aquatic to terrestrial species [5–7]. In terms of fish diseases, *A. hydrophila* can infect various ranges of species [8–10]. Among all the genomes publicly available on GenBank until 2022, *A. hydrophila* has been reported to have been isolated from dozens of fish species, such as goldfish, carp, snakehead, eel, tilapia, catfish, bluegill, large yellow croaker, sturgeon, and salmon as summarized in Table S1. *A. hydrophila* can be both a primary and secondary bacterial pathogen in fish, depending on the fish’s health conditions [8, 11]. It is well known as an opportunistic pathogen that can infect individuals under poor and stressed conditions. Due to these characteristics, outbreaks of *A. hydrophila* are used as indicators of poor water quality management and polluted environments. In addition, *A. hydrophila* can survive in a wide range of water temperatures (0–45 °C) [8, 12, 13]. *A. hydrophila* naturally confronts a variety of environments that require adaptation. These attributes have the potential to act as evolutionary drivers, allowing bacteria to endure prolonged exposure to various challenging environments and adapt accordingly. *A. hydrophila* harbor numerous extracellular proteins which are known to be associated with environmental adaptability [14]. In response to varying environmental conditions, bacteria undergo adaptations by modulating gene expression and composition, leading to the emergence of diverse phenotypic characteristics, including virulence [4, 15, 16]. Not only *Aeromonas* but there are many cases that the genotypic differences affected significantly phenotypic characteristics such as pathogenicity and pH resistance in other fish bacterial pathogens [17, 18]. Alterations in the bacterial genome can exert profound effects on the intricate transcriptional network, subsequently influencing the associated phenotypic traits [19]. This suggests an increasing importance in acquiring comprehensive insights into the genotypic characteristics of *A. hydrophila* that are associated with its transcriptomic and/or phenotypic traits. Despite its significance, the integration of genomic and transcriptomic data in *A. hydrophila* across diverse evolutionary contexts has remained insufficiently addressed up until now.

In recent years, massive genomic and transcriptomic results have been released, and all these works contribute to the understanding of the physiological characteristics of fish bacterial pathogens [20–27]. The significance of interpreting and analyzing massive and deep sequencing data is increasingly recognized in the exploration of novel insights into bacterial evolution and the dynamics of genetic content alteration [28–30]. The integration of omics data derived from independent studies holds significant potential in providing novel insights and generating substantial impact through the application of big data analysis in microbiological research [31]. However, each study employs different bioinformatics pipelines, strategies, and tools for individual specific purposes, thus posing a challenge in intuitively comparing results across different datasets.

Flagella are widely recognized for their crucial roles in motility, adhesion, and biofilm formation, which are directly associated with the growth and adaptation of bacteria in viscous environments or spaces [32–34]. The diversity of flagella subunits can influence the assembly, shape, size, and movement of flagella, which results in variations in functionality [35–37]. Given the fact that a single polar flagellum allows *A. hydrophila* to swim efficiently and move in diverse environments, facilitating its invasion of host cells, the understanding of genotypic and transcriptomic characteristics in the flagellin subunits can reveal the microbiological significance of flagellin in *A. hydrophila* [38, 39]. During the pan-genome and comparative genomic study, we have noticed that strains from terrestrial and aquatic environments tend to harbor different compositional patterns of flagellin subunits, known to possess unique and typical/atypical expression patterns and/or functionalities. Accordingly, this study manually aligned and processed *A. hydrophila* genome and/or transcriptome data using a standardized in-house pipeline. The main objective was to elucidate the genotypic and transcriptomic characteristics of *A. hydrophila* flagellin subunits that might greatly impact their phenotypic traits.

## Materials and Methods

### Strains and Genomic Data

In this study, *A. hydrophila* genomes with available sample histories, including information on isolation site, date, and source from the BioProject database, were utilized. Table S1 provides comprehensive information about the strains used in this study, including isolated year, country, host, and accession numbers in GenBank, RefSeq, BioProject, and BioSample. In total, 151 *A. hydrophila* strains from 18 countries between 1980 and 2022 were analyzed in this study. Average nucleotide identity (ANI) in all *A. hydrophila* genomes and six other *Aeromonas* species (*Aeromonas caviae*, *Aeromonas salmonicida*, *Aeromonas sobria*, *Aeromonas veronii*, *Aeromonas jandaei*, *Aeromonas dhakensis*) were calculated using FastANI (version 1.34) [40]. A heatmap and dendrogram were created using the “gplots” package in R (version 4.2.3), based on ANI results among strains [41]. The dendrogram analysis employed the “Euclidean” method for distance calculation and clustered using the “average” method. Based on the isolation source, all strains were categorized into either aquatic or terrestrial environments. Among the 151 strains, 108 were classified as originating from aquatic environments, while the remaining 43 were categorized as terrestrial strains (Table S1). Flagellin homologs in *A. hydrophila* were examined, and three subunits of flagellin genes, annotated as flagellin A (*flaA*), flagellin B (*flaB*), and flagellin (*fla*), were identified using the NCBI Prokaryotic Genome Annotation Pipeline [42]. The number and type of flagella genes varied among strains [43].

### Flagellin Homologs

The number of *flaA*, *flaB*, and *fla* gene copies from each strain was used for the dendrogram analysis using “ape” and “phytools” packages in R (version 4.2.3) [44–46]. Five distinct groups were identified based on the composition and number of flagellin homologs. This study named each strain named into *fla*(+ 1) to *fla*(+ 4) groups based on the number of flagellin copies. These groups encompassed strains with two to four copies of *fla* (abbreviated as *fla*(+ 2), *fla*(+ 3), or *fla*(+ 4)), strains with a combination of a single copy of *flaA* and *flaB* (abbreviated as *flaA*(+ 1)/*flaB*(+ 1)), and strains with a single copy each of the three flagellin subunits (abbreviated as *fla*(+ 1)/*flaA*(+ 1)/*flaB*(+ 1)). The relative proportion of the aquatic strains was determined using the following formula:

1 $$\mathrm R\mathrm e\mathrm l\mathrm a\mathrm t\mathrm i\mathrm v\mathrm e\;\mathrm p\mathrm r\mathrm o\mathrm p\mathrm o\mathrm r\mathrm t\mathrm i\mathrm o\mathrm n\;\mathrm o\mathrm f\;\mathrm s\mathrm t\mathrm r\mathrm a\mathrm i\mathrm n\mathrm s\;\mathrm f\mathrm r\mathrm o\mathrm m\;\mathrm a\mathrm q\mathrm u\mathrm a\mathrm t\mathrm i\mathrm c\;\mathrm e\mathrm n\mathrm v\mathrm i\mathrm r\mathrm o\mathrm n\mathrm m\mathrm e\mathrm n\mathrm t\;\left(\text{AE}\right)=\frac{\mathrm N\mathrm u\mathrm m\mathrm b\mathrm e\mathrm r\;\mathrm o\mathrm f\;\mathrm s\mathrm t\mathrm r\mathrm a\mathrm i\mathrm n\mathrm s\;\mathrm f\mathrm r\mathrm o\mathrm m\;\mathrm o\mathrm f\;\mathrm a\mathrm q\mathrm u\mathrm a\mathrm t\mathrm i\mathrm c\;\mathrm e\mathrm n\mathrm v\mathrm i\mathrm r\mathrm o\mathrm m\mathrm e\mathrm n\mathrm t\mathrm s\;\mathrm i\mathrm n\;\mathrm e\mathrm a\mathrm c\mathrm h\;\mathrm f\mathrm l\mathrm a\mathrm g\mathrm e\mathrm l\mathrm l\mathrm i\mathrm n\;\mathrm g\mathrm r\mathrm o\mathrm u\mathrm p}{\text{Total}\;\mathrm{nu}\mathrm m\mathrm b\mathrm e\mathrm r\;\mathrm o\mathrm f\;\mathrm s\mathrm t\mathrm r\mathrm a\mathrm i\mathrm n\mathrm s\;\mathrm f\mathrm r\mathrm o\mathrm m\;\mathrm a\mathrm q\mathrm u\mathrm a\mathrm t\mathrm i\mathrm c\;\mathrm e\mathrm n\mathrm v\mathrm i\mathrm r\mathrm o\mathrm n\mathrm m\mathrm e\mathrm n\mathrm t\;\left(n=108\right)}$$

2 $$\mathrm R\mathrm e\mathrm l\mathrm a\mathrm t\mathrm i\mathrm v\mathrm e\;\mathrm p\mathrm r\mathrm o\mathrm p\mathrm o\mathrm r\mathrm t\mathrm i\mathrm o\mathrm n\;\mathrm o\mathrm f\;\mathrm s\mathrm t\mathrm r\mathrm a\mathrm i\mathrm n\mathrm s\;\mathrm f\mathrm r\mathrm o\mathrm m\;\mathrm t\mathrm e\mathrm r\mathrm r\mathrm e\mathrm s\mathrm t\mathrm r\mathrm i\mathrm a\mathrm l\;\mathrm e\mathrm n\mathrm v\mathrm i\mathrm r\mathrm o\mathrm n\mathrm m\mathrm e\mathrm n\mathrm t\;\left(\text{TE}\right)=\frac{\mathrm N\mathrm u\mathrm m\mathrm b\mathrm e\mathrm r\;\mathrm o\mathrm f\;\mathrm s\mathrm t\mathrm r\mathrm a\mathrm i\mathrm n\mathrm s\;\mathrm f\mathrm r\mathrm o\mathrm m\;\mathrm o\mathrm f\;\mathrm t\mathrm e\mathrm r\mathrm r\mathrm e\mathrm s\mathrm t\mathrm r\mathrm i\mathrm a\mathrm l\;\mathrm e\mathrm n\mathrm v\mathrm i\mathrm r\mathrm o\mathrm m\mathrm e\mathrm n\mathrm t\mathrm s\;\mathrm i\mathrm n\;\mathrm e\mathrm a\mathrm c\mathrm h\;\mathrm f\mathrm l\mathrm a\mathrm g\mathrm e\mathrm l\mathrm l\mathrm i\mathrm n\;\mathrm g\mathrm r\mathrm o\mathrm u\mathrm p}{\text{Total}\;\mathrm{nu}\mathrm m\mathrm b\mathrm e\mathrm r\;\mathrm o\mathrm f\;\mathrm s\mathrm t\mathrm r\mathrm a\mathrm i\mathrm n\mathrm s\;\mathrm f\mathrm r\mathrm o\mathrm m\;\mathrm t\mathrm e\mathrm r\mathrm r\mathrm e\mathrm s\mathrm t\mathrm r\mathrm i\mathrm a\mathrm l\;\mathrm e\mathrm n\mathrm v\mathrm i\mathrm r\mathrm o\mathrm n\mathrm m\mathrm e\mathrm n\mathrm t\;\left(n=43\right)}$$

3 $$\begin{array}{c}\mathrm{Relative\;proportion\;of\;aquatic\;strains\;in\;each\;flagellin\;group}=\\\frac{\text{AE}}{(\text{AE}+\text{TE})}\end{array}$$

Chi-square analysis was performed using the relative proportion of strains from the aquatic environment (AE) and relative proportion of strains from terrestrial environment (TE) using the “gmodels” package in R [47]. *p*-value less than 0.05 was considered a statistical significance.

### Phylogenetic Analysis of Flagellin Homologs

The sequences of all flagellin homologs were obtained from genomic coding sequences and GFF/GTF files. Approximately, 400 flagellin nucleotide sequences were utilized for phylogenetic analysis using Nextstrain [48]. Briefly, all flagellin sequences are grouped by type of flagellin composition, isolation year, and isolation site. Also, “sequences-per-group,” which specifies the maximum number of sequences per group, was assigned to 50. All sequences were aligned by the option of “–file-gap” without reference sequence for constructing the phylogenetic tree. Time-resolved tree was generated through the option of “data-confidence﻿”, “coalescent = opt﻿”, and “data-inference = marginal﻿”. The trait, ancestral sequence inference, and mutation sites were annotated and profiled using the “traits﻿”, “ancestral﻿”, and “translate” commands. The phylogenetic results were then visualized in auspice using the “nextstrain view” command line.

### Flagellin Expression Level and Global Transcriptome

The investigation into the transcriptomic characteristics of various flagellin genes involved the selection of five strains (23-C-23, ATCC7966, NJ-35, SCV-1, and WCX23). These strains were chosen given their composition of different flagellin genes in their genome and availability in open databases. At least one of selected five strains has been confirmed to harbor the *fla*, *flaA*, and *flaB* genes. The flagellin gene and global bacterial transcriptomic expressions under ordinary cultural environments currently available in SRA (Sequence Read Archive) were profiled from five *A. hydrophila* strains using raw transcriptomic files (FastQ) [22–24]. Three to four biologically replicated FastQ files derived from the transcriptome of each *A. hydrophila* strain cultured under ordinary culturable conditions (e.g., tryptone soy or Luria–Bertani medium at 28 °C for ~ 1 day) were downloaded from SRA [20–24]. The strain WCX23 and 23-C-23 belong to *flaA*(+ 1)/*flaB*(+ 1) flagellin group, and ATCC7966 harbored single *flaA*, *flaB*, and *fla* gene in the genome (*flaA*(+ 1), *flaB*(+ 1), and *fla*(+ 1) group). On the other hand, NJ-35 and SCV-1 strain have three *fla* genes belonging to a flagellin *fla*(+ 3) group (Table 1).

**Table 1:** The information of strains used for transcriptomic study

Strain	Flagellin group	Label	SRA accessions	Culture media and incubation
23-C-23/ [20]	*flaA*(+ 1)/*flaB*(+ 1)	23-C-23_1 23-C-23_2 23-C-23_3	SRR13823239 SRR13823238 SRR13823237	23-C-23 strain was cultured in tryptone soy broth or agar (TSB or TSA) at 28 °C for ~ 24 h
ATCC7966/ [21] and accession (PRJNA668874)	*flaA*(+ 1)/*flaB*(+ 1)/ *fla*(+ 1)	ATCC7966_1 ATCC7966_2 ATCC7966_3 ATCC7966_4	SRR12825175 SRR12825182 SRR12825177 SRR4362845	ATCC7966 was cultured on Luria broth (LB) agar at 28 °C for 24 h or not identifiable
NJ-35/ [22, 23]	*fla*(+ 3)	NJ-35_1 NJ-35_2 NJ-35_3 NJ-35_4	SRR5894319	NJ-35 strain was incubated in tryptic soy broth at 28 °C for 24 h
			SRR11470407, SRR11470408 SRR11470411	NJ-35 wild-type strain was cultured in Luria–Bertani broth at 28 °C
SCV-1 (WT)/ [24]	*fla*(+ 3)	SCV-1_1 SCV-1_2 SCV-1_3	SRR15959704 SRR15959703 SRR15959702	SCV-1 strain was cultured in Luria–Bertani (LB) medium at 28 °C
WCX23/ [20]	*flaA*(+ 1)/*flaB*(+ 1)	WCX23_1 WCX23_2 WCX23_3	SRR13823236 SRR13823235 SRR13823234	WCX23 strain was cultured in tryptone soy broth or agar (TSB or TSA) at 28 °C for ~ 24 h

**Fig. 1:**
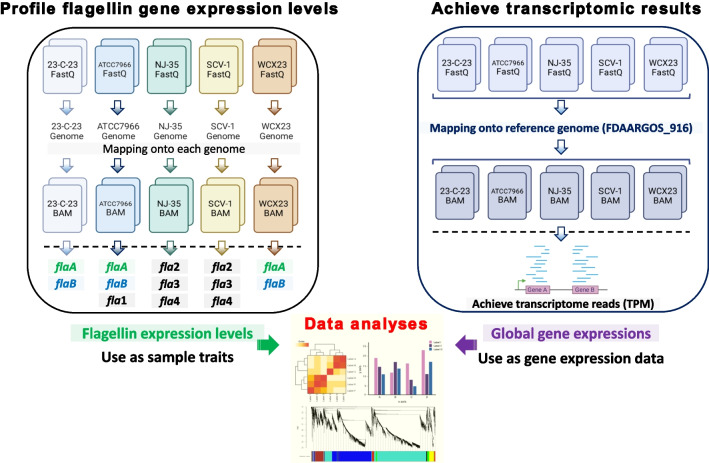
Schematic for profiling the expression of each flagellin gene and global gene expressions among the strains used in this study. The figure was drawn using BioRender (https://biorender.com/)

In this study, two distinct bioinformatics pipelines were utilized to assess the diverse levels of flagellin expressions across each strain, as well as to conduct a comparative analysis of the global transcriptome among the strains. To quantify the expression of various subunits of flagellin present in individual strains, transcriptomic reads were aligned to the genome of each strain (Fig. 1). In detail, FastQ files after trimming bad quality sequence reads below Q20 scores using Trimmomatic (Galaxy version 0.38.1) [49] were mapped onto each reference genome (WCX23; NZ_CP038463, 23-C-23; NZ_CP038465, ATCC7966; NZ_JAGDEM010000001, NJ-35; NZ_CP006870, and SCV-1; DANIKG010000001) using Bowtie2 with very sensitive end-to-end option (–very-sensitive) (Galaxy version 2.5.0 + galaxy0) [50, 51]. The transcriptome assembly, merge, and quantification were carried out using cufflinks (Galaxy version 2.2.1.3), cuffmerge (Galaxy version 0.0.5), and cuffdiff (Galaxy version 2.2.1.6) [52]. The FPKM (fragments per kilobase per million mapped fragments) value was validated to TPM (transcripts per million) value using below formula. TPM values of flagellin homologs from each sample were utilized for the level of flagellin gene expression as sample traits in this study. Welch’s *t*-test and Duncan’s multiple range test (analysis of variance; ANOVA) were carried out for the transcriptomic levels of each type of flagellin using an “aricolae” package in R (version 4.2.3) [53].

4 $$TPM= \frac{({\text{FPKM}}\;\mathrm{value\;at\;each\;gene\;}\times\;1000000)}{\mathrm{Sum\;of\;all\;transcriptome\;FPKM\;values}}$$

On the other hand, as for the global gene expression for the comparison among strains, all transcription reads were mapped onto one specific reference genome (FDAARGOS_916; NZ_CP065651). Except for the reference genome, the same pipeline was used as described above for obtaining TPM values (Fig. 1).

### Bioinformatics

#### Differentially Expressed Genes (DEGs)

Based on global transcriptome results, the differentially expressed genes between *flaA*(+ 1)/*flaB*(+ 1) (WCX23 and 23-C-23 strains) and *fla*(+ 3) (NJ-35 and SCV-1 strains) groups were observed. The *p*-value of each gene between flagellin groups was calculated using edgeR in R. The gene lower than *p*-value 1.14 × × 10^ −5^ from the Bonferroni adjustment (0.05/total number of genes observed in this study) was regarded as DEGs in this study. The DEGs were annotated and mapped onto Kyoto Encyclopedia of Genes and Genomes (KEGG) pathways [54].

#### Weighted Correlation Network Analysis (WGCNA)

The transcriptomic results from the strains that harbored *flaA* and *flaB* genes (WCX23, 23-C-23, and ATCC7966) were utilized for WGCNA. *FlaA* and *flaB* TPM results were used as sample traits. The signed network with the option of verbose = 3, merge cut height = 0.25, minimum module size = 30, and soft threshold power = 20 was generated to identify the gene cluster shared similar pattern of expression. Each module eigengene was used for correlation analysis with the expression of *flaA* and *flaB* to identify modules significantly correlated with their pattern of expression (*p*-value < 0.05). All genes belonging to significant modules were annotated using KEGG. Similarly, WGCNA was conducted under the same conditions as described above, utilizing NJ-35 and SCV-1 strains, which possess three *fla* genes (*fla-2*, *fla-3*, and *fla-4*) belonging to the *fla*(+ 3) group. All analyses and data visualization were carried out using “matrixStats﻿”, “impute﻿”, and “WGCNA” packages in R (version 4.2.3) [55].

#### Correlation Analysis

Correlation coefficients between sample traits (*flaA* and *flaB*) and all gene expressions were used for the clustering analysis. The heatmap with dendrogram was visualized using “RColorBrewer﻿”, “gplot﻿”, “pheatmap﻿”, and “d3heatmap” in R (version 4.2.3). Based on the pattern of correlation coefficients between all genes and *flaA* and *flaB*, eight gene clusters were divided. Based on the pattern of overall correlation coefficients with *flaA* and *flaB* in a cluster, positive coefficients were denoted with “ +﻿” , while negative coefficients were denoted with “ −﻿” . Gene clusters harbored both positive correlation coefficients in *flaA* and *flaB* (*flaA* + /*flaB* +), opposite direction of correlation coefficients between homologs (*flaA* + /*flaB*- or *flaA*-/*flaB* +), or both negative correlation coefficients (*flaA*-/*flaB*-) were annotated through KEGG pathways.

#### KEGG Pathway Enrichment

Genes in significant WGCNA modules and gene clusters were mapped onto KEGG pathways. Regarding the DEG results, genes showing significantly higher expression in the *flaA*(+ 1)/*flaB*(+ 1) groups compared to the *fla*(+ 3) groups were denoted as upregulated, while the reverse scenario was labeled as downregulated. The significance of up- and downregulated DEG numbers in each pathway was evaluated using the Chi-square analysis, and the pathway with a *p*-value less than 0.05 was visualized. Likewise, top five pathways enriched by genes from significant modules through WGCNA and gene clusters with the different patterns of *flaA* and *flaB* correlation coefficients were drawn by “EnrichmentMap” in Cytoscape 3.10.0 [56, 57]. The KEGG pathways relevant to human diseases were not used for the analyses in this study. As for the WGCNA and gene cluster analyses, the KEGG two-component system, which includes many genes and has relatively broad biological meaning and implications, was not included in data visualization.

## Results

### Average Nucleotide Identity (ANI)

In this study, ANI results of 151 genomes were in a range of 95.3555–99.9997%, with one exception in B11 strain. The ANI between the label *A. hydrophila* strain B11 and other *A. hydrophila* strains was between 93.2555 and 94.1305%. However, it exhibits a higher ANI with *A. dhakensis* (97.1336%) (Figure S1; Table S2). The ANI among five strains (WCX23, 23-C-23, ATCC7966, NJ-35, and SCV-1) utilized for transcriptomic analysis in this study ranged from 96.6255 to 99.9980% (Table S2). Among them, a high ANI sharing of over 99.9% was observed between the 23-C-23 and WCX23 strains, as well as between the NJ-35 and SCV-1 strains. Interestingly, among these five strains, those sharing high ANI values correspondingly belong to the same flagellin group (*flaA*(+ 1)/*flaB*(+ 1) and *fla*(+ 3)).

### The patterns of Flagellin Homolog Compositions and Phylogenetics in *A. hydrophila*

**Fig. 2:**
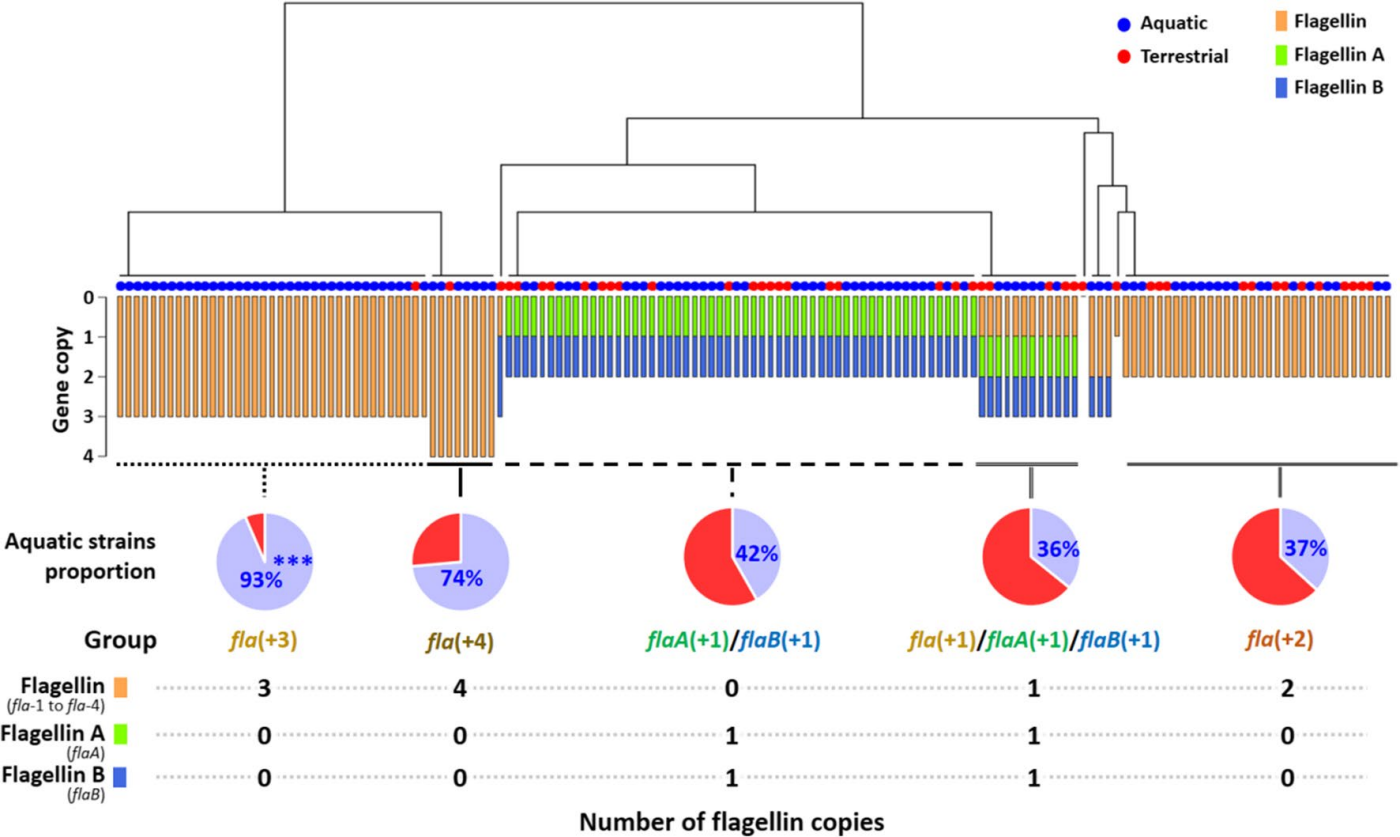
Dendrogram based on the type and number of flagellin genes by *Aeromonas hydrophila* strains. The blue and red circles indicate the isolated site of each strain (either aquatic or terrestrial environment). Bar color and length denote the type and number of flagellin genes. The relative proportion of strains originating from aquatic environment was described by the group sharing the same number of type of flagellin genes (*fla*(+3), *fla*(+4), *fla*(+1)/*flaB*(+1), *fla*(+1)/*flaA*(+1)/*flaB*(+1), and *fla*(+2)). Asterisks (***) indicates the significantly different number of aquatic strains compared to terrestrial strains in same flagellin group using Chi-square analysis (*p*-value < 0.001)

**Fig. 3:**
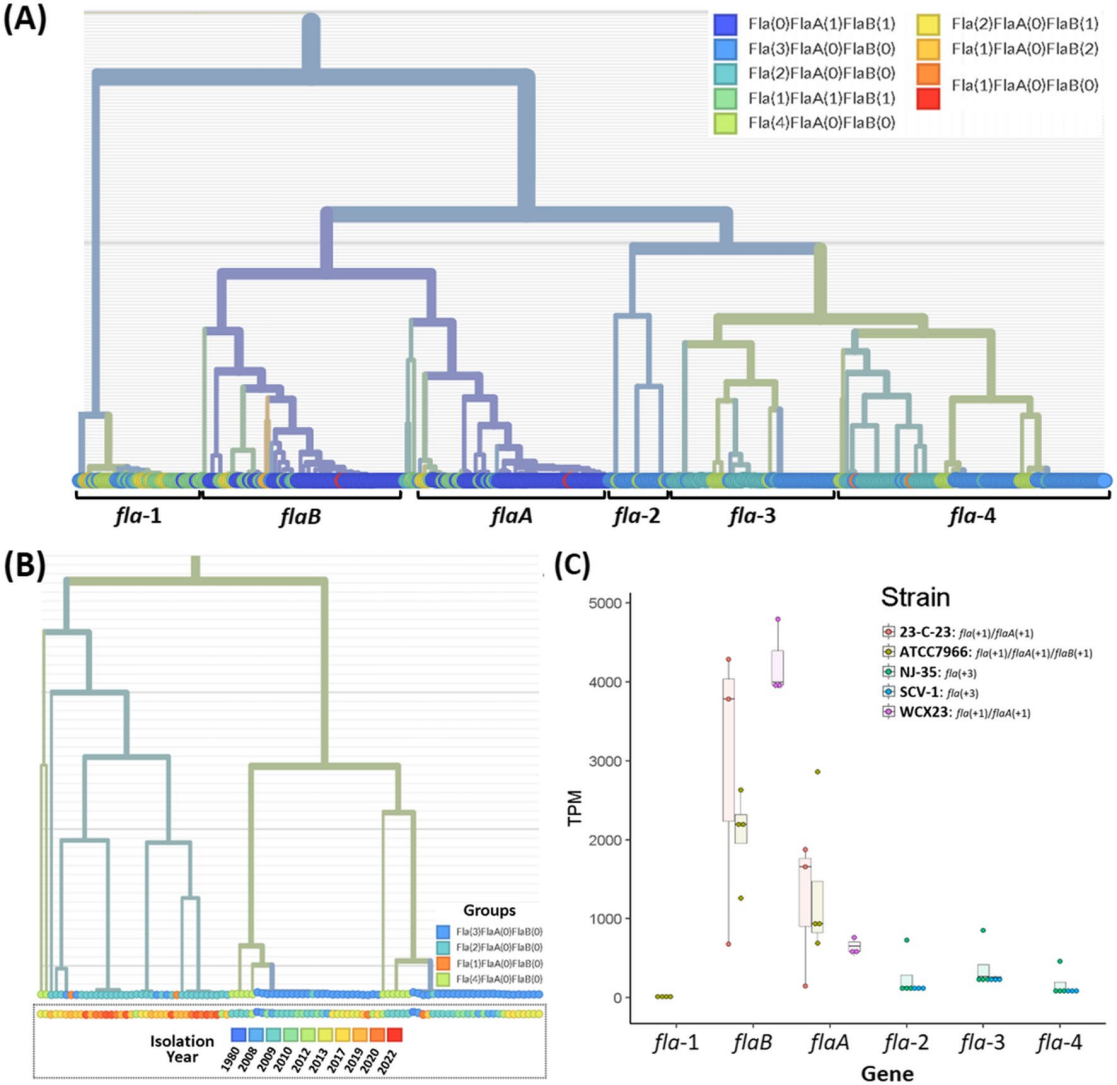
Phylogenetic tree of all flagellin genes in *Aeromonas hydrophila* observed in this study (**A**). Zoom in on the *fla*-4 clade with the label of isolation years (**B**). The transcription level of flagellin paralogs (*fla*-1, *flaB*, *flaA*, *fla*-2, *fla*-3, and *fla*-4) in 23-C-23, ATCC7966, NJ-35, SCV-1, and WCX23 strain based on transcripts per kilobase million (TPM) value (**C**). There was no statistically different expression between or among the strain within a same type of flagellin based on Welch’s *t*-test or one-way analysis of variance test using Duncan’s multiple comparison test

**Fig. 4:**
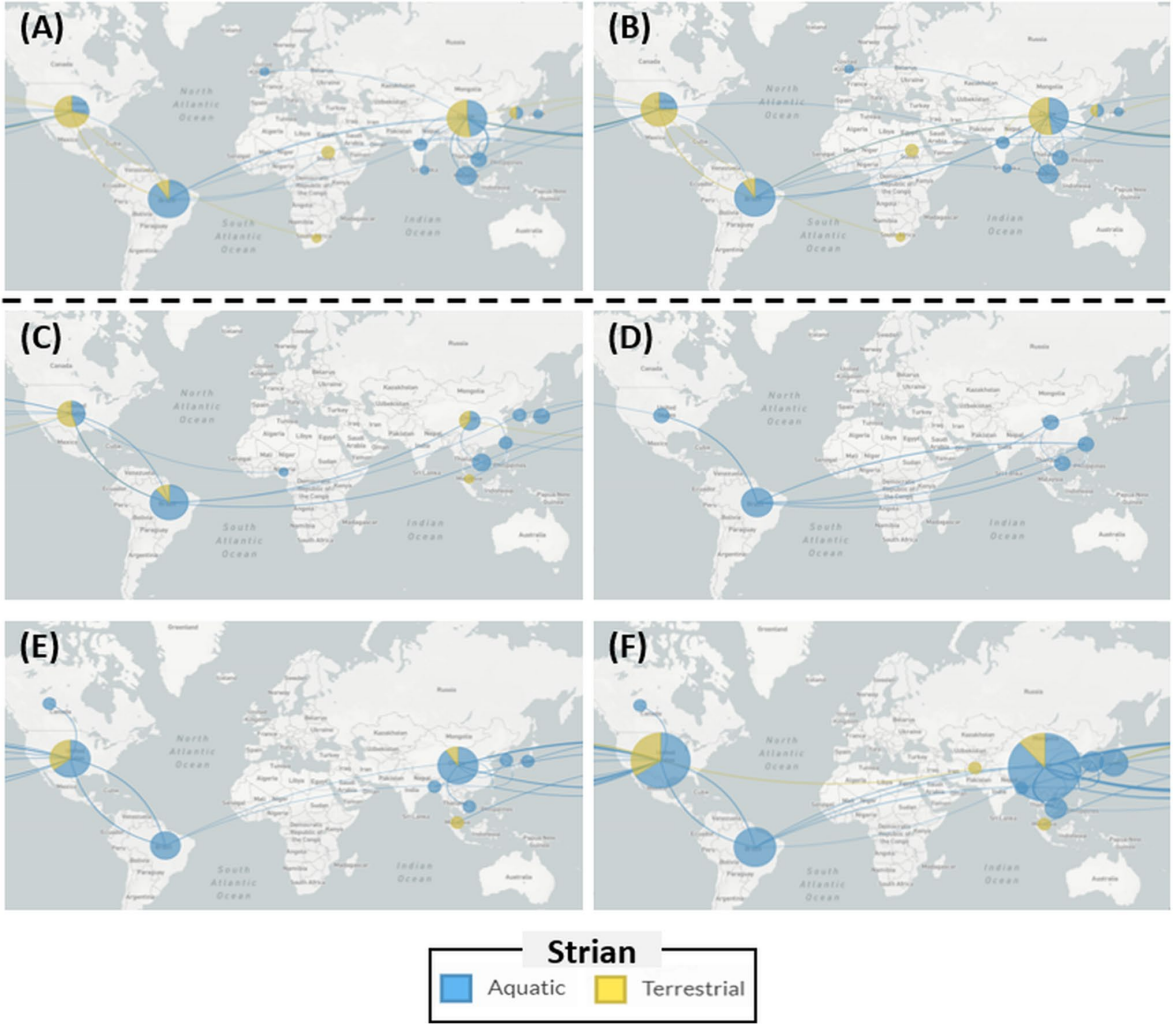
Phylogeographic analysis and distribution of aquatic and terrestrial strains in *flaA* (**A**), *flaB* (**B**), *fla*-1 (**C**), *fla*-2 (**D**), *fla*-3 (**E**), and *fla*-4 (**F**) genes

The number and type of flagellin genes in each strain were examined, revealing that 37 strains belonged to the *fla*(+ 3) group, 8 strains to the *fla*(+ 4) group, 56 strains to the *flaA*(+ 1)/*flaB*(+ 1) group, 12 strains to the *fla*(+ 1)/*flaA*(+ 1)/*flaB*(+ 1) group, 32 strains to the *fla*(+ 2) group, and the rest 6 strains that did not belong the majority. The strains harboring *flaA* and *flaB* were found to originate predominantly from terrestrial environments, whereas the *fla*(+ 3) and *fla*(+ 4) flagellin groups predominantly consisted of strains from aquatic environments. Specifically, the relative proportion of aquatic strains within the *fla*(+ 3) group was significantly higher compared to strains from terrestrial environments (Fig. 2). Based on phylogenetic analysis, the flagellin genes were classified into six subunits: four *fla* groups (named as *fla*-1, *fla*-2, *fla*-3, and *fla*-4, which were annotated same term but phylogenetically far distance flagellin), *flaA*, and *flaB* (Fig. 3). Interestingly, a notable observation was that the majority of strains did not possess a single type of flagellin gene, but instead exhibited a combination of different flagellin subunits. For instance, in the *fla*(+ 2) and *fla*(+ 3) flagellin groups, it was found that most strains harbored *fla*-1, *fla*-2, *fla*-3, and/or *fla*-4 genes, rather than multiple copies of the same flagellin type (Fig. 3A). The majority of strains belonging to the *fla*(+ 2), *fla*(+ 3), and *fla*(+ 4) groups possess at least one *fla-4* gene. However, there were slight phylogenetic differences observed between *fla*-4 genes from the *fla*(+ 2) and *fla*(+ 3) groups (Fig. 3A, B). Additionally, it was noted that *fla*(+ 2) strains tended to be isolated more recently compared to *fla*(+ 3) strains (Fig. 3B). In terms of gene expression levels, *flaB* and *flaA* exhibited the highest and second-highest expression levels among the seven flagellin homologs, respectively. In contrast, *fla*-2 to *fla*-4 displayed relatively similar lower levels of expression. Conversely, the *fla*-1 gene showed very low or no expression (Fig. 3C). The phylogeographic results revealed that a significant number of strains positive for *flaA* and *flaB* originated from terrestrial sources, particularly strains from China and the USA (Fig. 4). On the other hand, most *fla*-2 to *fla*-4 genes were derived from aquatic strains, and notably, no terrestrial strain was observed in *fla*-2. In contrast, *fla*-1, which displayed the most distant phylogenetic relationship with other flagellin genes, exhibited a higher proportion of terrestrial strains compared to *fla*-2 to *fla*-4 (Fig. 4).

### DEGs and Featured Pathways in the Flagellin Group

**Fig. 5:**
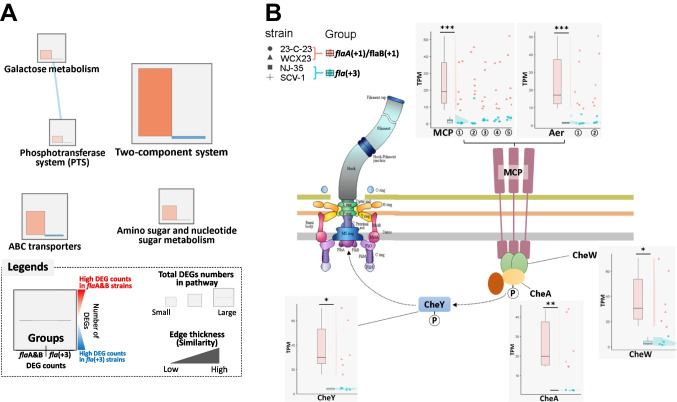
The KEGG enrichment analysis revealed a significantly different number of DEGs (differentially expressed genes) based on Chi-square analysis (*p*-value < 0.05) between *flaA*(+1)/*flaB*(+1) and *fla*(+3) strains (**A**). The two-component system relevant to flagellar motor switch adaptation based on the KEGG pathway for the prokaryote (**B**). The TPM level of relevant genes (*mcp*, *aer*, *cheY*, *cheA*, and *cheW*) was shown in raincloud plots. The expression of five homologous or paralogous *mcp* (Gene id: ①I6G73_RS10070, ②I6G73_RS19490, ③I6G73_RS04110, ④ I6G73_RS08330, and ⑤ I6G73_RS16810) and two *aer* (Gene id: ①I6G73_RS19930 and ②I6G73_RS05190) was shown as dot plots in accordance with different strains and groups. Asterisks (*p < 0.05, **p < 0.01, and ***p < 0.001) showed statistical difference between *flaA*(+1)/*flaB*(+1) and *fla*(+3) groups based on Welch two sample *t*-test

In total, 486 DEGs were identified between *flaA*(+ 1)/*flaB*(+ 1) and *fla*(+ 3) group, and 459 DEGs showed higher expression in *flaA*(+ 1)/*flaB*(+ 1) group. Five featured KEGG pathways, namely two-component system (ko02020), ABC transporters (ko02010), amino sugar and nucleotide sugar metabolism (ko00520), galactose metabolism (ko00052), and phosphotransferase system (PPTS) (ko02060), were identified as the most significantly different numbers of up- and downregulated DEGs across different flagellin groups. Notably, the *flaA*(+ 1)/*flaB*(+ 1) group exhibited a high number of upregulated DEGs in all five identified pathways (Fig. 5A). In particular, the two-component system pathway exhibited the highest number of DEGs among the identified pathways. Within this pathway, the flagellar motor switch adaptation system played a significant role, involving several DEGs such as methyl-accepting chemotaxis (*mcp*); aerotaxis receptor (*aer*); purine-binding chemotaxis protein (*cheW*); two-component system, chemotaxis family, sensor kinase (*cheA*); and two-component system, chemotaxis family, chemotaxis protein (*cheY*). In the *A. hydrophila* ASM162068 genome, five copies of *mcp* and two copies of *aer* were identified. The average gene expression levels of these genes were significantly higher in the *flaA*(+ 1)/*flaB*(+ 1) flagellin group. Similarly, the *flaA*(+ 1)/*flaB*(+ 1) group exhibited significantly higher TPM values in the genes *cheW*, *cheA*, and *cheY* compared to the *fla*(+ 3) group (Fig. 5B).

### Module Identification Correlated with Flagellin Expressions Through WGCNA

**Fig. 6:**
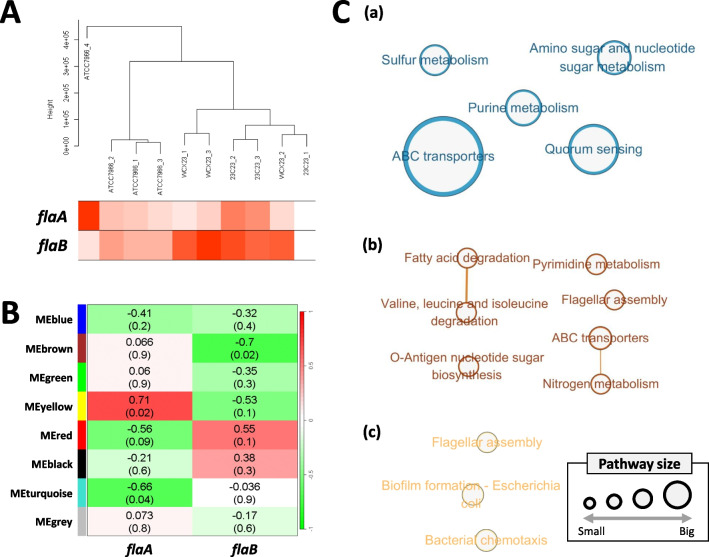
Clustering dendrogram with the *flaA* and *flaB* expression in each strain (**A**). The thicker red color denotes the higher *flaA* or *flaB* expression. The correlation between module eigengene and *flaA* or *flaB* expression (**B**). The highest enriched KEGG pathways in a turquoise (**C**-a), brown (**C**-b), and yellow module (**C**-c)

The dendrogram showed overall transcriptomic expression of ATCC7966, WCX23, and 23-C-23, along with the relative expression levels of *flaA* and *flaB* utilized in the WGCNA (Fig. 6A). In total, eight modules were identified, and the eigengene of yellow and turquoise modules was positively and negatively correlated with the expression of *flaA* (Figure S2; Fig. 6B). On the other hand, *flaB* showed only a negatively significant correlation with brown eigengene (Fig. 6B). In total, 2125, 366, and 188 genes were labeled and identified as the turquoise, brown, and yellow modules, respectively. Also, since two-component system was the pathway composed of the highest numbers belonging to each module, regardless of module’s significance, it was not visualized in this study. ABC transporters and quorum sensing are the pathways with high number of genes in turquoise modules. But, featured pathways in brown module were mainly linked to biomolecule degradation (e.g., fatty acid, valine, leucine, and isoleucine) and metabolism (e.g., pyrimidine and nitrogen metabolism). Although the yellow module consisted of a relatively small number of genes compared to others, these genes were found to be closely associated with flagellar assembly, biofilm formation, and bacterial chemotaxis. On the other hand, *fla*-2, *fla*-3, and *fla*-4 expression patterns were almost identical among samples, and their correlation coefficient values with six detected modules were also similar (Figure S3).

### Correlation Analysis Between All Gene and *flaA/flaB* Expressions

**Fig. 7:**
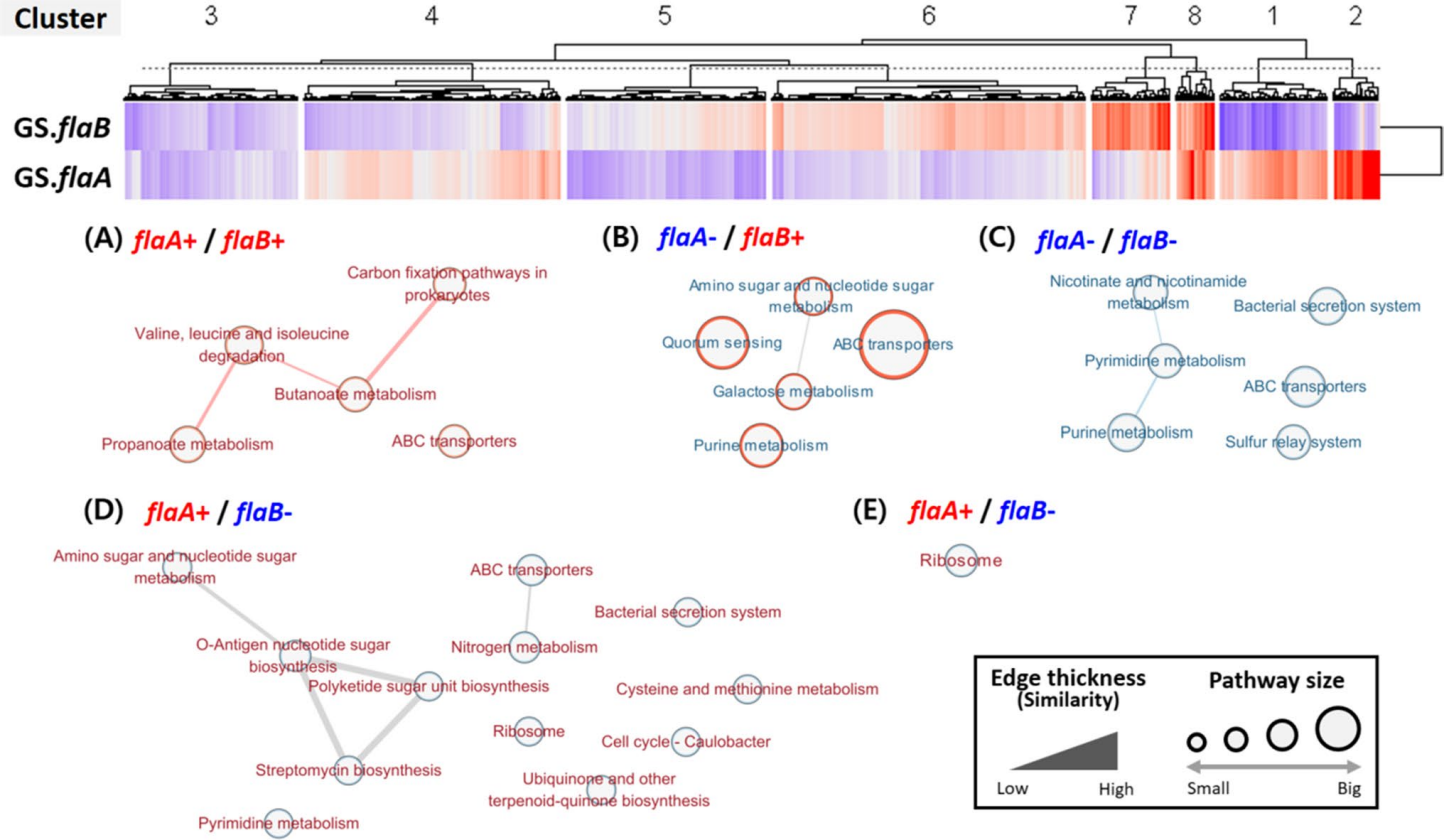
Dendrogram with heatmap based on the correlation coefficient between flagellin gene (*flaA* or *flaB*) and other gene expressions among *A. hydrophila* strains. Enriched KEGG pathways from the gene cluster 8 (**A**), cluster 6 (**B**), cluster 3 (**C**), cluster 1 (**D**), and cluster 2 (**E**)

The correlation coefficients between all gene expressions and flagellin genes (*flaA* and *flaB*) are shown in Fig. 7. Eight clusters (cluster 1–cluster 8) were identified based on correlation coefficient values between *flaA* and *flaB*. The top five pathways, each consisting of more than three genes from each cluster, were selected. Cluster 8, with both high expressions in *flaA* and *flaB* (*flaA* + /*flaB* +), comprised the genes that positively correlated with the expression of both *flaA* and *flaB* and were relevant to the propanoate and butanoate metabolism, valine, and leucine and isoleucine degradation. On the other hand, all genes that have both *flaA* and *flaB* negative correlation coefficients belonging to cluster 3, with both downregulations in *flaA* and *flaB*, (*flaA*-/*flaB*-), were mainly mapped onto the pyrimidine and purine metabolism, bacterial secretion system, etc. Clusters 1 and 2 showed a positive *flaA* but negative *flaB* correlation coefficient (*flaA* + /*flaB*-), and ribosome was the only pathway shared between clusters. Although many KEGG pathways share the same number of genes, relatively, many pathways were visualized in cluster 2, O-antigen nucleotide sugar biosynthesis, nitrogen metabolism, and ABC transporters were the highest pathways (Fig. 7). On the other hand, quorum sensing and ABC transporters were one of the featured pathways in cluster 6, composed of genes with negative *flaA* but positive *flaB* correlation coefficients (Fig. 7).

## Discussion

In this study, we maximized the use of genomic and transcriptomic data to investigate the characteristics of different subunits of flagellin in *A. hydrophila*. This study incorporated comparative genomics analyses and conducted comprehensive profiling of transcriptomic responses across five representative strains of *A. hydrophila*. These strains harbored various copies of flagellin genes with characteristics that have yet to be clearly elucidated in their genomes. To assess the expression levels of individual flagellin genes, we mapped the transcriptomic data to their respective reference genomes. On the other hand, in examining the overall and global transcriptomic responses across strains, we analyzed the transcriptomic data by aligning it to a single reference genome. This approach facilitates a thorough investigation of the global transcriptomic expression across strains, enabling the preservation of diverse flagellin expression profiles within each genome. Additionally, it helps to the identification of correlated genes associated with flagellin expression.

Most of the *A. hydrophila* genomes exhibited a high ANI of over 95%; however, the B11 strain showed ANI values in the range of 93 to 94%, which are lower than the ANI comparison with *A. dhakensis* (~  97%). The mislabeling issue of *Aeromonas* species in the GenBank database has been raised previously. Beaz-Hidalgo et al. [58] found that mislabeled *A. hydrophila* genomes in the GenBank, which had ANI values ranging from 93.70 to 93.84% with the *A. hydrophila* ML09-119 strain, actually belonged to *A. dhakensis*. Taken together, although the B11 strain has been labeled and utilized as *A. hydrophila*, it actually belongs to *A. dhakensis*. Considering that *A.* *dhakensis* has been documented as a highly virulent species capable of causing mortality in both humans and fish [59–61], further studies on the classification and understanding of *A. dhakensis* are necessary. However, the B11 strain did not harbor any distinctiveness in the pattern of flagellin subunit composition compared to other *A. hydrophila* strains. Among the five strains used for the transcriptomics study, the 23-C-23 and WCX23 strains, as well as the NJ-35 and SCV-1 strains, shared a high ANI of over 99.9%. Given that former studies [62, 63] reported that the isolation of clonal related *A. hydrophila* strains sharing high ANI (>  99%) in the fish, the strains over 99% ANI in this study may be genotypically clonal-related strains. 

Flagella play a crucial role not only in the motility and virulence of bacteria but also in their evolutionary adaptation to diverse environmental conditions [64, 65]. Six flagellin clusters were phylogenetically identified among the flagellin sequences obtained from 151 *A. hydrophila* genomes, including *flaA*, *flaB*, and four additional flagellin. Notably, this study revealed significant phylogenetic variations among four *fla* genes annotated with the same term. Consequently, these flagellins were arbitrarily designated as *fla*-1 to *fla*-4. In general, the different subunits of flagellin composition can affect the swimming behaviors of bacteria [66]. Kühn et al. [67] investigated the phenotypic characteristics of the different assemblies of *flaA* and *flaB* in *Shewanella putrefaciens*, and *flaA* mutant strain (Δ*flaA*) and *flaB* mutant strains (Δ*flaB*) showed completely different patterns of swimming and colony structure. An impaired swimming capacity with significantly reduced filament length was observed in the Δ*flaB* strain, while Δ*flaA* strain did not exhibit substantial differences compared to the wild-type strains. Conversely, in the case of *Helicobacter pylori*, the Δ*flaA* strains showed a loss of motility, whereas the Δ*flaB* mutants retained their motility [68]. This indicates that the significance of *flaA* and *flaB* concerning bacterial motility may vary depending on the species and could be directly associated with bacterial motility. Interestingly, this study revealed that the majority of *fla*(+ 3) strains were derived from aquatic sources rather than terrestrial environments. In contrast, *A. hydrophila* strains in the *flaA*(+ 1)/*flaB*(+ 1) and *fla*(+ 1)/*flaA*(+ 1)/*flaB*(+ 1) groups tend to be more frequently isolated from terrestrial environments and animals. In *Shewanella putrefaciens*, diverse fractions of cells demonstrating screw motion in a high-viscosity environment were found to be influenced by the specific flagellin type [67]. Similarly, in the case of *Aeromonas hydrophila*, the observed variations in flagellin subunits harbored by different strains are likely to be a consequence of adaptive responses to distinct environmental challenges. Likewise, Nakamura and Minamino [36] suggested that *flaA* can be the principal component in soil bacteria such as *Sinorhizobium meliloti* and *Rhizobium lupine*. These results highlight the potential impact of flagellin diversity on the motility behavior of microorganisms and the adaptive significance of such variations in coping with varying environmental conditions.

In *Aeromonas* species, the sequence and genetic diversity in flagellin genes have greatly influenced the activity of biofilm formation, pathogenicity, and swimming motility [69]. Miyagi et al. [69] investigated genetic diversity in *flaA* in *A. hydrophila*, and the phylogenetic clusters were clearly divided in accordance with the type of origins, either clinical or environmental basis. The different level of motility and biofilm activity observed by *flaA* clusters indicated the genetic changes in the flagellin gene could drive different phenotypic characteristics. Given that flagella-relevant proteins, which directly exposed to outer environments, they can exhibit high variability due to external conditions. Based on the massive sequence information in *Vibrio* *harveyi*, the group incubated in seawater at 30 °C revealed a possibility that changes the genetic mutation in the flagellar relevant gene (putative flagellar motor switch protein) [29]. This study also verified some flagellin genes, for example *fla*-4, were clustered with the time of isolation (isolation year). This implied that the status of flagellin genes in *A. hydrophila* is not static but highly mutable and changeable, which can lead to environmental adaptation. However, the composition or nucleotide sequence of flagellin subunits did not show a significantly different pattern by country, not like subunits of environments such as aquatic and terrestrial traits.

The expression level of flagellin genes in ordinary cultural environments differed significantly depending on the flagellin type. *FlaA* and *flaB* showed relatively high expression, while *fla*-1 to *fla*-4 was not highly expressed like *flaA* and *flaB*. Interestingly, the strains belonging to the *flaA*(+ 1)/*flaB*(+ 1) group comprised many upregulated DEGs relevant to two-component system. Among many subordinated two-component systems, bacterial chemotaxis relevant to the flagellar motor switch adaptation pathway is featured and enriched subordinated systems in *flaA*(+ 1)/*flaB*(+ 1) group. In general, bacteria utilize chemotaxis to control their movement toward favorable environments by detecting the levels of chemoeffectors (e.g., attractants and repellents) through chemoreceptors [70]. The level of chemoeffectors can be detected by *mcp* and *aer*, and these signals were delivered through the *cheW* and *cheA* [71]. The level of autophosphorylated *cheA* is greatly influenced by signal transduction, which affects the stimulation of *cheY* by phosphorylation. Ultimately, phosphorylated *cheY* interacts with the flagellar motor and switches the direction of rotation in the flagellar [70, 72]. The high expression of *flaA* and *flaB*, along with the elevated expression of chemoreceptors and response regulator proteins (*cheW*, *cheA*, and *cheY*), might indicate that *flaA*(+ 1)/*flaB*(+ 1) group may have more robust flagella activity and/or movement, compared to the *fla*(+ 3) group. In addition, given that more than half number of strains in *flaA*(+ 1)/*flaB*(+ 1) group originated from terrestrial habitats where high variations of temperature and humidity were observed [73], it has been speculated that *A. hydrophila* can evolve and develop the type of flagellin fitting into the facing environment. Also, bacterial flagellins are known to the important epitope that causes the strong immuno-stimulatory response through toll-like receptor in aquatic animals [64, 74, 75]. Gao et al. [64] investigated that the level of immuno-stimulation effects varies depending on the subunits of flagellin (*flaA*, *flaB*, *flaC*, *flaD*, and *flaE*). This implies that the composition of flagellin genes in *A. hydrophila* could also have a significant impact on infection and prognosis.

In general, the construction of relationship networks based on the pattern of co-expression through pairwise measurement could be a robust approach for investigating biologically meaningful pathways. WGCNA, as the more advanced method, has received great attention to clarify the relationship between multidimensional results and featured sample traits [55, 76]. In this study, we applied the WGCNA method to identify the gene clusters that are highly correlated with *flaA* and *flaB* expression. *FlaA* and *flaB* were not typically co-expressed in *A. hydrophila*. For example, the yellow module, which positively correlated with the expression pattern of *flaA* but showed no significance with *flaB*, consisted of genes related to flagellar assembly, bacterial chemotaxis, and biofilm formation. On the other hand, a brown module, which showed a negative correlation with *flaB* expression but lacked significance with *flaA*, harbored the same flagellar assembly pathway. This does not necessarily imply that *flaA* and *flaB* have opposing functions in flagella assembly, but rather indicates that they do not share identical functions and characteristics. While both *flaA* and *flaB* may be associated with the flagellar assembly pathway, they may act in potentially different capacities or manners. Mohari et al. [77] investigated that *Agrobacterium* *tumefaciens* have several subunits of flagellin from *flaA* to *flaD*. *FlaA* in *A. tumefaciens* showed the highest importance that affected flagellin motility and functions, but others (*flaB*–*flaD*) had accessory functions [77]. Likewise, transcriptional expression of *flaA* and *flaB* in *H. pylori* is controlled by different sigma factors (ς28 and ς54), which suggests that both flagellin were regulated in a different timely manner [68, 78, 79]. Considering gene clusters or gene correlation analysis, it is a high possibility that *flaA* is probably associated with biofilm formation, bacterial chemotaxis, and bacterial secretion systems. Besides, *flaB* might be more associated with quorum sensing activities. But further studies are necessary to confirm this hypothesis.

## Conclusion

This study conducted the first comprehensive investigation of hundreds of *A. hydrophila* genomes to profile flagellin subunits and clusters. We identified *flaA*, *flaB*, and four other phylogenetically different units of flagellin (*fla*-1 to *fla*-4). The composition of flagellin genes in *A. hydrophila* genomes exhibited significant diversity among strains, with a predominant presence of 3 to 4 *fla* subunits (*fla*(+ 3) and *fla*(+ 4) groups) originating from aquatic environments. In contrast, many terrestrial strains tended to harbor different flagellin subunits (*flaA* and *flaB*) in their genomes. Through multiple transcriptomic analyses conducted on strains with different flagellin subunits compositions under ordinary culture conditions, this study demonstrated significantly higher expression of *flaA* and *flaB* compared to the other four flagellins. Furthermore, genes associated with flagellin motor switch adaptation, controlling bacteria chemotaxis movement, showed significantly higher expression in the *flaA*(+ 1)/*flaB*( +) group compared to *fla*(+ 3). While further studies are necessary to elucidate the specific functions of each flagellin subunit, the pattern of flagellin composition and expression level found in this study provide valuable insights into the understanding of the role of flagellin and function and the adaptive mechanisms of microorganisms in diverse environments.

### Supplementary Information

Below is the link to the electronic supplementary material.

Supplementary figures (DOCX 548 KB)

Table S1 (XLSX 23 KB)

Table S2 (XLSX 199 KB)

## Data Availability

The sequence data utilized in this study are accessible in the Sequence Read Archive (SRA) under the accession number specified in Table 1 and Table S1.
