# Supplementary material for: Genomic and Transcriptomic Diversification of Flagellin Genes Provides Insight into Environmental Adaptation and Phylogeographic Characteristics in Aeromonas hydrophila
Source: Microb Ecol. 2024 May 2;87:65. doi: 10.1007/s00248-024-02373-4 (PMC11065939; doi:10.1007/s00248-024-02373-4)
Supplement: Supplementary file 1 [file 248_2024_2373_MOESM1_ESM.docx]

**Figure S1.** Heatmap with dendrogram of all genomes used in this study and five other *Aeromonas* species (*Aeromonas caviae*, *Aeromonas salmonicida*, *Aeromonas sobria*, *Aeromonas veronii*, *Aeromonas jandaei*, and *Aeromonas dhakensis*) based on average nucleotide identity (ANI). The red or blue color in the heatmap indicated the percent identity of ANI between two genomes.

**Figure S2.** Dendrogram based on the expression patterns of all genes among samples. Each gene is categorized into one of eight module colors.

**Figure S3.** Clustering dendrogram of 7 samples from NJ-35 and SCV-1 strains with individual *fla2*, *fla3*, and *fla4* expression level. The correlation between module eigengene and *fla2*, *fla3*, and *fla4* expression (B). Dendrogram based on the expression patterns of all genes among samples. Each gene is categorized into one of six module colors (C).
